# Utility of mass spectrometry and artificial intelligence for differentiating primary lung adenocarcinoma and colorectal metastatic pulmonary tumor

**DOI:** 10.1111/1759-7714.14246

**Published:** 2021-11-23

**Authors:** Wataru Shigeeda, Ryuichi Yosihimura, Yuji Fujita, Hidekazu Saiki, Hiroyuki Deguchi, Makoto Tomoyasu, Satoshi Kudo, Yuka Kaneko, Hironaga Kanno, Yoshihiro Inoue, Hajime Saito

**Affiliations:** ^1^ Department of Thoracic Surgery Iwate Medical University Iwate Japan; ^2^ Division of Critical Care Medicine, Department of Critical Care, Disaster and General Medicine Iwate Medical University Iwate Japan; ^3^ Shimadzu Corporation Kyoto Japan

**Keywords:** mass spectrometry, metastatic pulmonary tumor, pulmonary adenocarcinoma

## Abstract

**Background:**

Rapid intraoperative diagnosis for unconfirmed pulmonary tumor is extremely important for determining the optimal surgical procedure (lobectomy or sublobar resection). Attempts to diagnose malignant tumors using mass spectrometry (MS) have recently been described. This study evaluated the usefulness of MS and artificial intelligence (AI) for differentiating primary lung adenocarcinoma (PLAC) and colorectal metastatic pulmonary tumor.

**Methods:**

Pulmonary samples from 40 patients who underwent pulmonary resection for PLAC (20 tumors, 20 normal lungs) or pulmonary metastases originating from colorectal metastatic pulmonary tumor (CRMPT) (20 tumors, 20 normal lungs) were collected and analyzed retrospectively by probe electrospray ionization‐MS. AI using random forest (RF) algorithms was employed to evaluate the accuracy of each combination.

**Results:**

The accuracy of the machine learning algorithm applied using RF to distinguish malignant tumor (PLAC or CRMPT) from normal lung was 100%. The algorithms offered 97.2% accuracy in differentiating PLAC and CRMPT.

**Conclusions:**

MS combined with an AI system demonstrated high accuracy not only for differentiating cancer from normal tissue, but also for differentiating between PLAC and CRMPT with a short working time. This method shows potential for application as a support tool facilitating rapid intraoperative diagnosis to determine the surgical procedure for pulmonary resection.

## Graphical abstract

Rapid intraoperative diagnosis for unconfirmed lung tumor is extremely important determining the surgical procedure. This study evaluated the usefulness of mass spectrometry (MS) for differentiating primary lung adenocarcinoma (PLAC) and colorectal metastatic pulmonary tumor (CRMPT). MS demonstrated high accuracy for differentiating not only cancer from normal tissue, but also PLAC and CRMPT with a short working time, suggesting the potential for application to rapid intraoperative diagnosis.

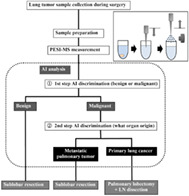

## INTRODUCTION

The lungs show a high frequency of developing metastases from malignant tumors originating in other organs, such as renal cell carcinoma, colorectal carcinoma, and breast cancer, and resection of metastatic lung tumors is actively performed.^1^ Rapid intraoperative diagnosis is required in cases involving resection of lung tumors with an unconfirmed diagnosis. In rapid diagnosis, both the distinction between benign and malignant lesions and the distinction between primary lung cancer and metastatic pulmonary tumor are extremely important for determining the most appropriate surgical procedure. This is because pulmonary lobectomy is the standard procedure for primary lung cancer, whereas sublobar resection (minimal resection preferably since wedge resection is standard; if not, pulmonary segmentectomy) as a reduction surgery is the basic procedure for metastatic pulmonary tumor.^2^ Since the resected lung volume and the lost pulmonary function differ significantly between lobectomy and sublobar resection, accurate pathological diagnosis is extremely important from the perspective of postoperative pulmonary hypofunction and decreased quality of life (QOL). However, in clinical practice, peripheral pulmonary tumors are diagnosed preoperatively in only about 50% of cases,^3^ and there are still many cases in which surgery is performed with unconfirmed preoperative diagnosis. Rapid intraoperative diagnosis is thus an absolutely essential tool during pulmonary resection.

Intraoperative rapid pathological diagnosis is currently performed under a relatively poor environment using frozen sections of coarse tissue slices, and differences are sometimes seen between the results of rapid diagnosis and the final histopathological diagnosis obtained from permanent specimens. This is unavoidable due to the limitation of modern medical technology, but represents a clinical problem. For example, if a primary lung cancer is diagnosed as metastatic pulmonary tumor intraoperatively, additional two‐stage residual completion lobectomy may be required as a radical resection at a later date, representing very invasive surgery for the patient.^4^, ^5^ Conversely, if a metastatic pulmonary tumor is diagnosed as primary lung cancer intraoperatively, excessive pulmonary lobectomy will be performed. Especially for elderly patients with low reserve pulmonary capacity, this will negatively impact pulmonary function and may lead to the need for home oxygen therapy. Subsequent QOL is likely to decline and even continued treatment of the originated primary organ malignancy may well be hindered. Given this background, the development of intraoperative rapid diagnosis methods offering higher accuracy within a short time is desired.

Currently, promising studies such as rapid immunohistochemical staining (R‐IHC) have been carried out for rapid intraoperative diagnosis.^6^ This method takes about 20–30 min, which is acceptable, but further reductions in time are desired to shorten the surgical and anesthesia times and reduce invasiveness for patients. Furthermore, the diagnosis is still very difficult for some metastatic pulmonary tumors of the same histological type. In recent years, attempts to diagnose malignant tumors such renal cell carcinoma^7^ and hepatocellular carcinoma^8^ using probe electrospray ionization (PESI)‐mass spectrometry (MS) have been reported.^9^, ^10^, ^11^, ^12^ Those previous studies have identified that some lipid substances are key to discrimination, while others are not,^7^, ^8^, ^9^, ^10^, ^11^, ^12^ but the discrimination itself is considered both possible and extremely useful for clinical applications. The advantage of this PESI‐MS is that diagnostic support can be performed within a few minutes, and has the potential to allow discrimination even in cases where morphological discrimination is difficult. The present study tried to estimate the usefulness of MS and artificial intelligence (AI) for differentiating between normal lung tissue, primary lung adenocarcinoma (PLAC) and colorectal metastatic pulmonary tumor (CRMPT).

## METHODS

### Patient selection

We reviewed medical records for this retrospective cohort study. Participants comprised consecutive patients with PLAC or metastatic pulmonary tumor who underwent radical pulmonary resection between October 2019 and March 2021 in the Department of Thoracic Surgery at Iwate Medical University. This study was approved by the institutional review board at Iwate Medical University, and the need for informed consent was waived based on the retrospective design (permit number: MH2019‐068).

Surgical indications for complete video‐assisted thoracoscopic surgery (c‐VATS) lobectomy comprise the concept of a thoracoscopically resectable lesion, covering almost 95% of surgical patients in our institute. All patients underwent complete preoperative pulmonary evaluation. Pulmonary function was tested at our institute using a spirometer (CHESTAC 8800; CHEST M.I., Tokyo, Japan) according to the standards of the American Thoracic Society.^13^ Vital capacity (VC) and forced expiratory volume in 1 s (FEV_1_) were measured preoperatively in patients within 1 month before surgery. Patients with chronic obstructive pulmonary disease (COPD) received bronchodilator therapy for at least 4 weeks preoperatively, and smoking was stopped for at least 8 weeks before surgery. Cases with tumor diameter less than 10 mm or ground‐glass opacity nodules (GGNs) on preoperative images were excluded from this study to ensure sufficient tissue samples could be obtained.

**FIGURE 1:**
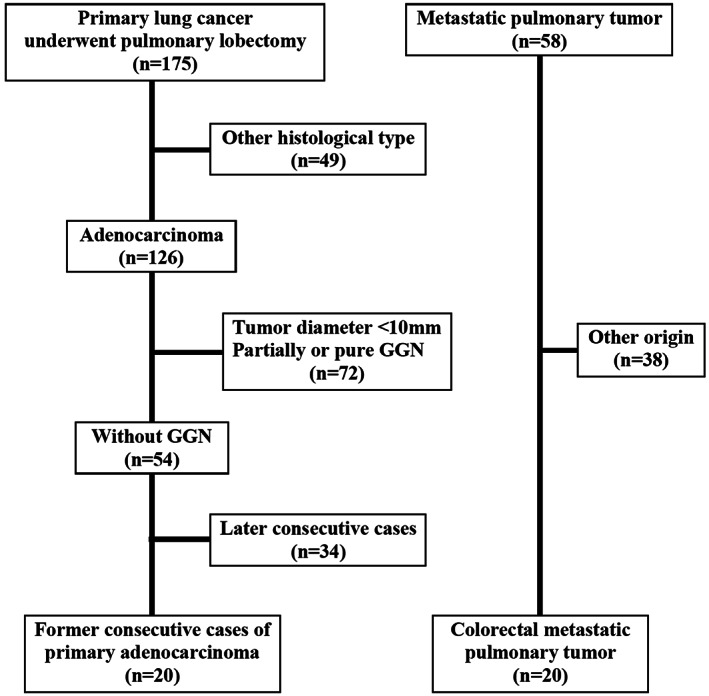
Patient selection in this study

Patients who had received preoperative chemotherapy or radiation to the primary lung cancer were excluded from this study. A final total of 40 patients (20 cases each of both primary lung cancer and metastatic pulmonary tumor) met the selection criteria (Figure 1).

### Surgical procedures

Pulmonary resection was performed under general anesthesia with a double‐lumen endotracheal tube for single‐lung ventilation. The affected lung was deflated as soon as the pleural space was opened, and deflation was maintained throughout most of the operative period. The patient was placed in the lateral decubitus position. Pulmonary resection by c‐VATS was performed via 3 ports under monitor vision only in all cases in this study. Wedge resection was basically performed for metastatic pulmonary tumors. If the tumor was small and was predicted to be difficult to identify during surgery, a CT‐guided hook wire was placed on the morning just before surgery.^14^ For cases in which wedge resection proved difficult, segmentectomy was performed without lymph node dissection. Pulmonary lobectomy and complete systematic lymph node dissection were performed in all primary lung cancer cases. After completing the procedure, the sealing test was performed before wound closure and confirmed during reinflation of the affected lung. A chest tube (Blake, 19‐Fr; Ethicon) was placed from the fifth intercostal trocar to the apex.

### Sample collection

Fresh tumor samples (5 × 5 × 5 mm) were taken from resected pulmonary tissues macroscopically judged as representing the neoplastic lesion and normal lung parenchyma, respectively, and were immediately deep frozen (−80°C) until analysis. Differentiation between primary lung cancer and metastatic pulmonary tumor from colorectal carcinoma was made by final pathological diagnosis, including immunohistochemistry.

### Measurement with MS and machine learning

**FIGURE 2:**
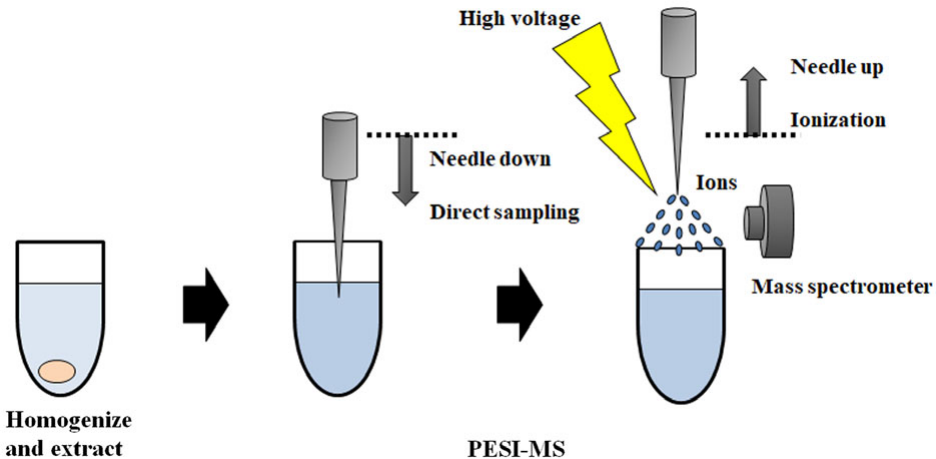
The procedure for sample analysis by probe electrospray ionization mass spectrometry (PESI‐MS)

PESI‐MS (DPiMS^‐^2020; Shimadzu Corp.) was applied for patients in whom the PLAC, CRMPT and normal lung parenchyma were analyzed, respectively (Figure 2). Briefly, for preparation of each sample before PESI‐MS, a 2‐mm diameter piece of tissue was cut with a scalpel and homogenized with a disposable tissue grinder (Pestle tube; Argos Technologies) in 100 μl of ethanol/water (50:50). Ten microliters of the homogenized solution was dispensed in the solvent drip position of the sample plate (Shimadzu Corp.) to perform PESI‐MS. Analyses were performed in positive ion mode with acquisition in full‐scan mode for 1 min in the range of mass‐to‐charge ratio (m/z) of 10–2000. The probe needle with a tip was moved downward to touch the sample solution and then upward to apply high voltage (2.45 kV) for electrospray ionization. This movement was repeated, and generated ions were introduced into the mass spectrometer. Mass spectra were acquired in full‐scan continuum mode (peak profile) and representative information for all mass peaks was exported using LabSolutions software (ver. 5.99SP2; Shimadzu Corp.). Each tissue sample was analyzed for 1 min in order to obtain sufficient mass spectra to maximize the signal‐to‐noise ratio and maintain an optimal ionization process, preventing loss of signal caused by PESI needle contamination.

Each 1‐min sample acquisition was arbitrarily divided into fragments of 10 s each. Mass spectra were averaged for every acquisition fragment and six mass spectra from one sample were exported for machine learning. AI was applied using a random forest (RF) discrimination algorithm for machine learning and to build the classification. Statistical validation and evaluation of RF models were performed using the cross‐validation method. Of the spectra derived from each sample, 70% were used to create the discrimination model, then tested against the others used for machine learning. The accuracy of these statistical models was evaluated in terms of concordance with classification by the pathologist. All processes from sample preparation to measurement and provision of results were completed within 4 min (Table 1).

**TABLE 1:** All processes from sample preparation to analysis

Process	Time
Sample preparation	
Cut the sample for tissue grinder	1 min
Homogenize the sample	1 min
Setting sample to PESI‐MS	30 s
MS measurement	1 min
AI analysis	30 s
Total	4 min

### Statistical analysis

JMP version 12.2.0 statistical software (SAS Institute) and Python version 3.6.8 (The Python Software Foundation), in which all discrimination algorithms including multivariate partial least‐squares discriminant analysis (PLS‐DA) and RF were implemented, were used for all statistical analyses. Groups were compared using the Pearson's chi‐square test or Wilcoxon's rank‐sum test. Differences between groups were considered significant at the level of *p* < 0.05. Continuous data are expressed as mean ± standard deviation. Categorical data are expressed as counts and proportions.

## RESULTS

A total of 40 patients who underwent pulmonary resection for PLAC (20 tumors, 20 normal lung tissues) or metastatic pulmonary tumor originating from CRMPT (20 tumors, 20 normal lung tissues) were analyzed retrospectively.

The clinical characteristics of the study population are summarized in Table 2. For eligible patients, no significant differences in age, gender, body mass index, or Brinkman index were evident between groups. Compared with the CRMPT group, the PLAC group showed a significantly higher frequency of patients with diabetes mellitus (30% vs. 0%, *p* < 0.009) and a significantly higher maximum tumor diameter (36.7 ± 12.4 mm vs. 14.7 ± 8.2 mm, *p* < 0.001), respectively.

**TABLE 2:** Clinical background and tumor characteristics of all patients who underwent pulmonary resection

	All patients		*p*‐value
	PLAC	CRMPT	
	(*n* = 20)	(*n* = 20)	
Age (years)	72.7 ± 5.4	71.4 ± 8.7	0.989
Gender			
Male	8 (40.0)	10 (50.0)	0.530
Female	12 (60.0)	10 (50.0)	
BMI (kg/m^2^)	23.5 ± 3.4	23.5 ± 4.0	0.892
Brinkman Index	421.5 ± 511.3	215.2 ± 341.7	0.457
Underlying disease			
COPD	5 (25.0)	5 (25.0)	1.000
Interstitial pneumonia	2 (10.0)	0 (0.0)	0.152
Antithrombotic therapy	0 (0.0)	3 (15.0)	0.075
Diabetes mellitus	6 (30.0)	0 (0.0)	0.009*
Preoperative pulmonary function			
VC (ml)	3101.0 ± 595.0	3099.5 ± 788.0	0.850
%VC (%)	111.0 ± 12.4	104.1 ± 12.0	0.102
FEV_1_ (ml)	2259.0 ± 400.4	2247.0 ± 480.6	0.695
%FEV_1_ (%)	105.0 ± 15.4	101.5 ± 19.5	0.402
%D_LCO_ (%)	103.5 ± 21.2	116.9 ± 28.1	0.092
CEA (ng/ml)	11.9 ± 27.9	6.8 ± 8.5	0.441
Pathological staging of colorectal cancer			
I	NA	2 (10.0)	NA
II	NA	5 (25.0)	
III	NA	9 (45.0)	
IV	NA	4 (20.0)	
Chemotherapy before pulmonary resection	NA	9 (45.0)	NA
Timing of pulmonary metastasis			
Synchronous	NA	2 (10.0)	NA
Metachronous	NA	18 (90.0)	
Procedure			
Lobectomy	20 (100.0)	4 (20.0)	NA
Segmentectomy	0 (0.0)	4 (20.0)	
Wedge resection	0 (0.0)	12 (60.0)	
Maximum tumor diameter (mm)	36.7 ± 12.4	14.7 ± 8.2	<0.001*
Lymph node metastasis			
N0	13 (65.0)	NA	NA
N1	5 (25.0)	NA	
N2	2 (10.0)	NA	
*EGFR* mutation			
Non	10 (50.0)	NA	NA
Exon 19 deletion	5 (25.0)		
Exon 21 L858R	5 (25.0)		

*Note*: *p < 0.05 versus PLAC group.

**FIGURE 3:**
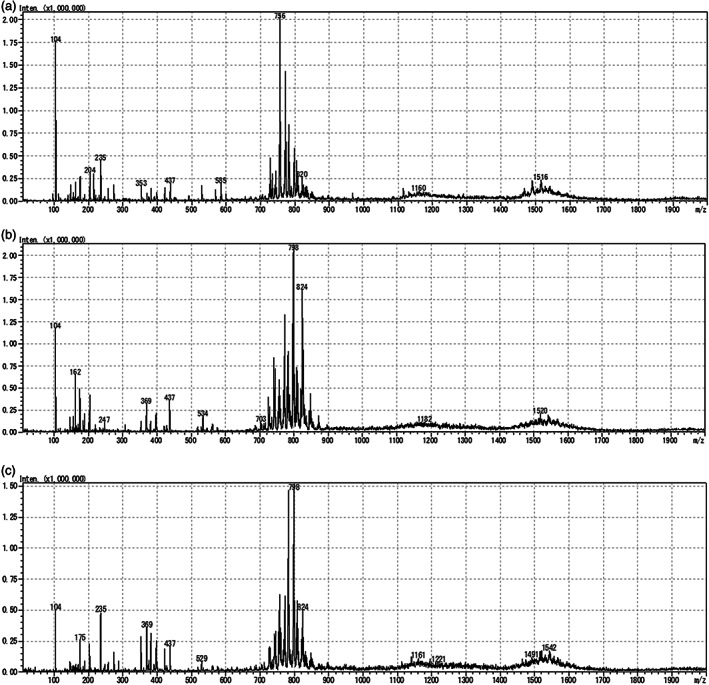
Representative full‐scan mass spectra, averaged from 10‐s acquisition fragments, from normal lung tissue (a), PLAC (b), and CRMPT (c)

**FIGURE 4:**
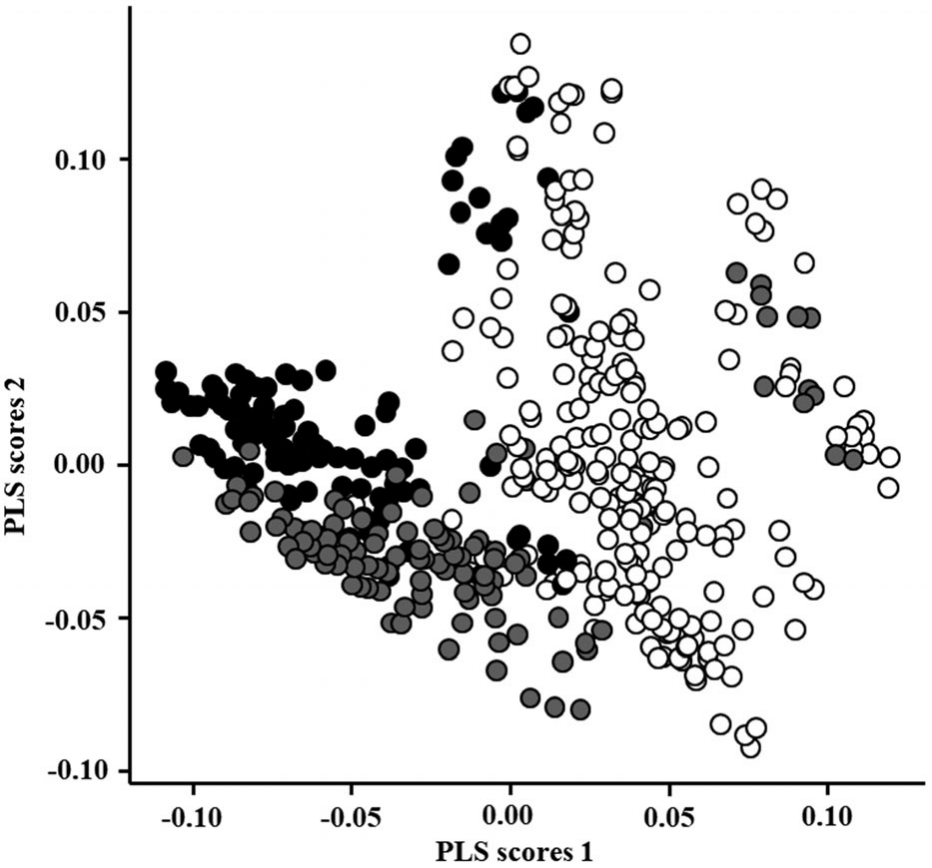
Discriminant analysis score plot of primary lung adenocarcinoma (PLAD, closed gray circles), colorectal metastatic pulmonary tumor (CRMPT, closed black circles) and non‐tumor lung tissue (open circles)

Figure 3 shows representative full‐scan mass spectra, averaged from 10‐s acquisition fragments, from normal lung tissue (a), PLAC (b), and CRMPT (c). Of the spectra derived from each sample, 70% were used to create the discrimination model, then tested against the others used for machine learning. We applied multivariate statistical analysis to test whether these datasets were separate from each other. PLS‐DA showed good separation of malignant tumor from normal lung tissue, and also of CRMPT from PLAC, although some overlapping data points from both groups remained (Figure 4).

**FIGURE 5:**
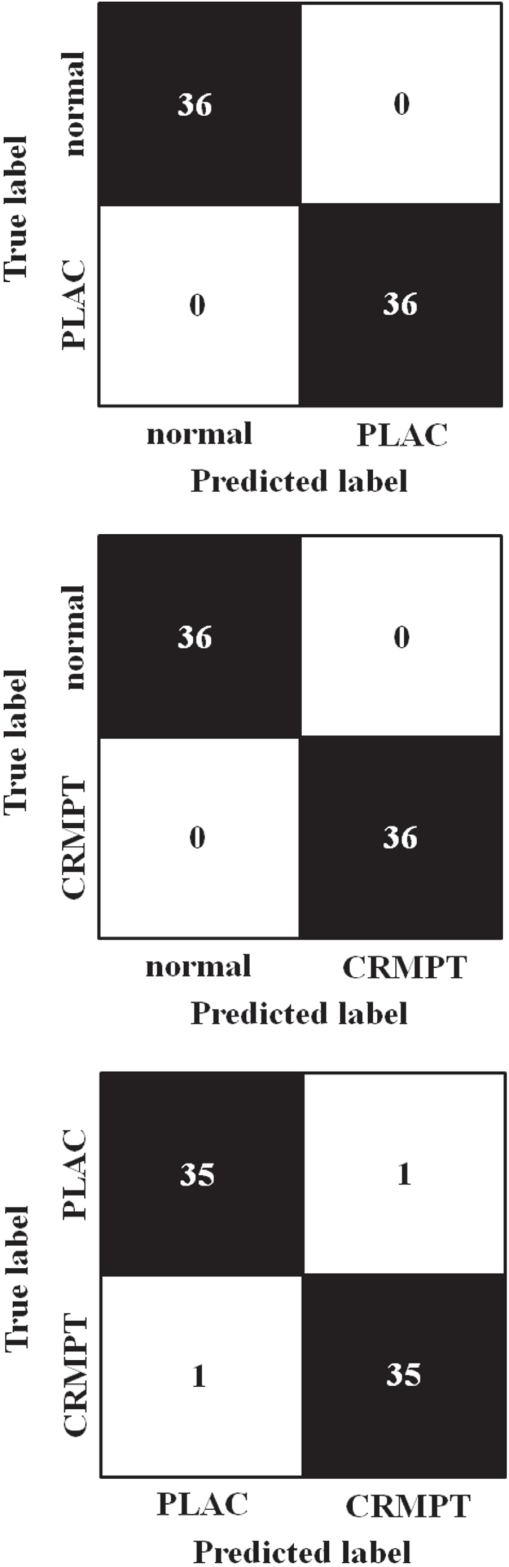
Confusion matrix of our random forest (RF) model showing true‐positive and true‐negative prediction, to distinguish the following combinations: PLAC versus normal lung tissue (a), CRMPT versus normal lung tissue (b), and PLAC versus CRMPT (c), respectively. True values were obtained from histopathological examination, while predicted values were obtained from the RF model

Machine learning algorithms were then applied RF, to distinguish the following combinations (Figure 5): PLAC versus normal lung tissues (a); CRMPT versus normal lung tissues (b); and PLAC versus CRMPT (c). The results showed 100% accuracy for distinguishing PLAC from normal lung tissues using 72 spectra (72/72 spectra), and also for distinguishing CRMPT from normal lung tissues using 72 spectra (72/72 spectra) (Table 3). Furthermore, for distinguishing PLAC and CRMPT, the 72 spectra analyzed showed just one false positive and one false negative each, so the algorithm yielded 97.2% accuracy (70/72 spectra). Although the number of cases was small, RF discrimination by AI was considered to correct for some overlapping dots seen in PLS‐DA by learning, thus producing more accurate results.

**TABLE 3:** A machine‐learning algorithm was applied using RF to distinguish the following combinations

	Accuracy (%)
PLAC vs. normal	100
CRMPT vs. normal	100
PLAC vs. CRMPT	97.2

## DISCUSSION

This study was conducted to demonstrate that MS with AI can be used to distinguish between normal lung and malignant tissues, and to distinguish between PLAC and CRPMT with high accuracy. Furthermore, measurement using MS followed by analysis using AI can obtain highly accuracy diagnosis within just a few minutes, and has potential for application as a support tool for rapid intraoperative diagnosis in actual clinical practice.

In pulmonary resection, situations in which intraoperative diagnosis influences the surgical procedure are relatively frequent in clinical practice. This is because the standard procedure for primary lung cancer is lobectomy, and the basic procedure for metastatic pulmonary tumor and benign disease is sublobar resection (minimal resection is preferred, since wedge resection is standard; if this is not feasible, then pulmonary segmentectomy is used), which is a reduction surgery.^2^ Obtaining an accurate diagnosis within a short time is thus imperative for determining the appropriate resection range within a limited operative time to preserve minimal invasiveness. Diagnosis using conventional frozen sections takes about 30 min, and morphological evaluation is sometimes difficult due to the relatively poor environment such as coarsely ground frozen sections. To overcome this problem, techniques such as R‐IHC have been developed and research into clinical applications is ongoing,^6^ but about 20 min is still needed to reach a diagnosis and the types of antibodies available can be limited.

**FIGURE 6:**
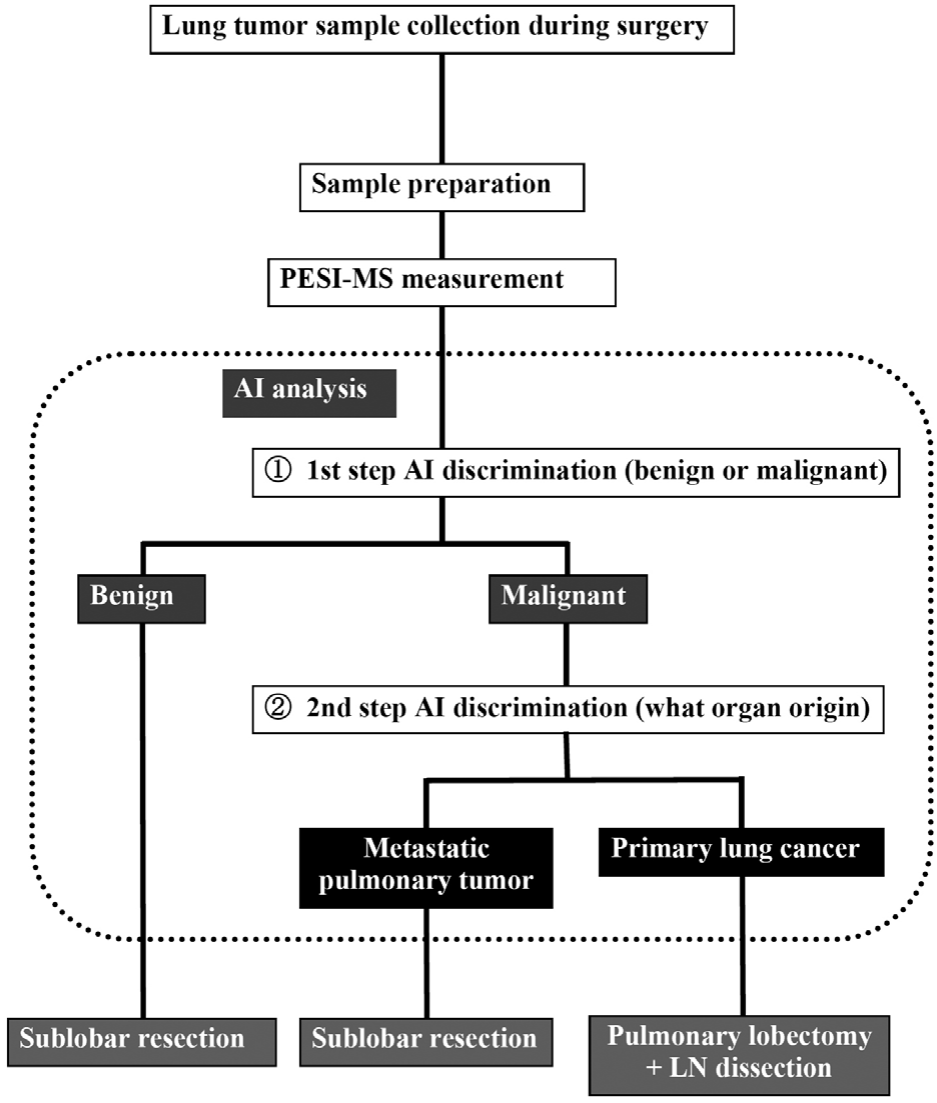
Each step for intraoperative diagnosis of pulmonary tumor using combined MS and AI in a two‐step discrimination (1: whether benign or malignant; and 2: if malignant, which is the organ of origin). All diagnosis steps were completed within about 4 min

In contrast, our MS‐AI combination method offers the advantage of allowing discrimination within about 4 min for the entire process from sample preparation to judgment. Moreover, highly accurate discrimination can be achieved within a short time using this two‐step discrimination (1: whether the lesion is benign or malignant; 2: if malignant, what is the organ of origin). Furthermore, if many types of sample data for metastatic pulmonary tumors from other organs are learned in advance, multiple diseases can be distinguished from just one sample with the addition of only few seconds (Figure 6). MS does not require the addition of reagents such as expensive antibodies or limited collected tissue samples, even in the differentiation of multiple diseases, and is thus advantageous in terms of cost, time, labor, and tissue sample requirements. In addition, no specialized knowledge or training is required and the results are extremely objective. Diagnostic support systems using MS‐AI thus have the potential to be very helpful in conventional rapid intraoperative pathological diagnosis systems. Another feature of these processes is that a very small amount of sample still yields highly accurate diagnosis. In this regard, the method may be applicable to the diagnosis of minute specimens such as from bronchoscopic examination of pulmonary tissues.

Rapid intraoperative diagnosis is often used to confirm the absence of residual cancer in the excisional surgical margin, to determine whether a tumor is malignant, or to confirm the histological type. The method of PESI‐MS is suitable for determining whether the cancer is localized at the probe puncture site, since the method involves ionizing the droplets generated when the probe is puncture into the tissue. PESI‐MS has thus been suggested to be useful in confirming the intraoperative resected surgical margin to check for cancer remnants.^11^, ^12^ However, since pulmonary tissue has the characteristic of being nonuniform, obtaining an accurate diagnosis from measurement of only one point puncture is difficult. Therefore, we can obtain highly accurate results by homogenizing the tissue to elute the tissue components into solution, inserting a probe into the resulting liquid, and analyzing the ionized droplet component by MS. This approach is considered useful for diagnosis of lymph node metastasis in which normal tissue and cancer cells are mixed heterogeneously coexist, and the method has been applied to the rapid diagnosis of regional lymph node metastasis in aggressive reduction surgery for early lung cancer.^15^

Actually, R‐IHC offers an excellent method of rapid diagnosis that is currently being studied for clinical application. However, some limitations need to be considered, especially the difficulty in distinguishing between thyroid transcription factor‐1 (TTF‐1)‐positive CRMPT and PLAC.^6^, ^16^, ^17^ Furthermore, distinguishing between primary lung squamous carcinoma and pulmonary metastasis of squamous cell carcinoma from another organ (e.g., head and neck carcinoma) by IHC is still difficult, even in the final pathological diagnosis. MS research will hopefully have the potential to solve these issues in the future.

In this study, our method demonstrated good separation of malignant tumor from normal lung tissue, and of CRMPT from PLAC, but some overlap such as false negatives remained. PLS‐DA showed relatively good separation of each group, while some overlap of both groups remained. Overlapping areas, especially false‐negative areas, are important in clinical practice. The background of patients showing false‐negative cases was examined. False‐negative results for CRMPT were found in two cases, both of which were metachronous recurrences. Postoperative adjuvant chemotherapy was performed after radical surgery for colorectal carcinoma in both cases. The cause remains unclear, but it is possible that chemotherapy might have some effect. Two false‐negative cases of PLAC were also seen, and both cases showed a mucinous adenocarcinoma.

It is possible that false negative may have occurred due to the characteristic of high proportion of mucinous benign compartments in adenocarcinoma. If those above is the reason, it could be solved by identifying biomarkers in the future and narrowing down the measurement window. However, the most likely cause was considered to be the small number of cases in this study, including false‐positive cases. One of the characteristics of AI learning is that accuracy increases with more learning. Many more cases should therefore be accumulated to improve accuracy in the future.

Many studies have been investigating specific biomarkers to discriminate between normal cells and cancer cells, and many previous reports have described lipid metabolites that might be useful as biomarkers.^18^, ^19^ Specifically, such reports have been made for renal cell cancer,^7^ liver,^8^ breast cancer,^10^ and colorectal cancer.^12^ Regarding lung cancer, several lipid metabolites have been reported as candidates in sputum analysis,^20^ but definitive biomarkers that include other organ malignancies have not yet been clarified. In this study, our system could not specify the metabolites from which the m/z was derived for each identification of PLAC, CRMT, and normal lung. However, peak m/z values in this study were suggested to be derived from lipid profiles which consistent with previous reports from other organs.^7^, ^8^, ^10^, ^12^ Our system does not deviate from the molecular prediction method, while focusing on the fingerprints of responsible molecules.

The present investigation had several limitations, including the retrospective design and the small number of patients from a single institution. Also, cases of GGN on PLAC were excluded from this study to ensure sufficient tissue samples were obtained. However, the results suggest that even GGN cases may be sufficiently differentiated. To further improve the accuracy of discrimination models, spectra from more cases need to be accumulated. Furthermore, continuing this research will clarify the metabolite molecules affecting discrimination prediction. If the metabolite molecules derived from m/z values used for identification can be determined, those biomarkers could be applied to the detection of cancer recurrence and cancer screening in the future. We plan to examine these m/z values in detail using MS/MS in the near future.

This MS‐AI system is expected to be applicable to rapid intraoperative diagnosis as a supporting tool improving accuracy and speed. If this method is clinically applied, accurate diagnosis in just 4 min may be achievable, shortening the time for surgery and anesthesia and facilitating reduced surgical invasiveness. Furthermore, excessive pulmonary resection and resurgery could be largely prevented, contributing to improvements in QOL, postoperative prognosis and medical economy.

In conclusion, MS combined with an AI system offered highly accurate differentiation of cancer from normal tissue, and of PLAC from CRMPT, with only a short working time. This method has the potential to be applied as a support tool for rapid intraoperative diagnosis during pulmonary resection.

## CONFLICT OF INTEREST

The authors declare that there are no conflicts of interest. This research received no specific grant from any funding agency in the public, commercial, or not‐for‐profit sectors.
